# Basic Chemical Composition, Antioxidant Activity and Selected Polyphenolic Compounds Profile in Garlic Leaves and Bulbs Collected at Various Stages of Development

**DOI:** 10.3390/molecules28186653

**Published:** 2023-09-16

**Authors:** Joanna Skoczylas, Elżbieta Jędrszczyk, Kinga Dziadek, Ewa Dacewicz, Aneta Kopeć

**Affiliations:** 1 Department of Human Nutrition, Faculty of Food Technology, University of Agriculture in Krakow, Balicka 122, 31-149 Kraków, Poland; joannaskoczylas7@gmail.com (J.S.); kinga.dziadek@urk.edu.pl (K.D.); 2 Department of Horticulture, Faculty of Biotechnology and Horticulture, University of Agriculture in Krakow, al. 29 Listopada 54, 31-425 Kraków, Poland; elzbieta.jedrszczyk@urk.edu.pl; 3 Department of Sanitary Engineering and Water Management, Faculty of Environmental Engineering and Land Surveying, University of Agriculture in Kraków, Adam Mickiewicz Ave. 24/28, 30-059 Kraków, Poland; ewa.wasik@urk.edu.pl

**Keywords:** *Allium sativum* L., time of harvest, proximate composition, cultivar, flavonoids, phenolic acids

## Abstract

Garlic is commonly used as vegetable or spice and as a herb in folklore as well as traditional medicine in many countries. The current study aimed to compare the chemical composition, antioxidant activity, and the content of selected polyphenolic compounds in cloves as well as leaves of winter garlic plants of the Harnaś and Ornak cultivars, which are of Polish origin. Garlic was grown from cloves for three years (2018–2020) in the experimental field of the University of Agriculture in Krakow, Poland. The research material was harvested on three dates: May; June, unripe garlic plants; and in July, the plant at full maturity. The content of vitamin C in the fresh material was determined. The proximate analysis was determined in the freeze-dried plants of garlic, and the total carbohydrate content was calculated. The antioxidant activity and the content of selected polyphenolic compounds were also determined. Garlic cloves showed a higher content of dry matter, and total carbohydrates than the leaves of garlic plants. On the other hand, in the leaves, a significantly higher content of protein, total fat and ash were observed. Additionally, garlic leaves were characterised by a higher content of vitamin C, total polyphenols, and a higher antioxidant activity than garlic cloves. The leaves of young garlic plants from the May harvest were distinguished by a higher content of these compounds. The dominant phenolic compounds were catechin and epicatechin. The leaves of young plants can be a valuable source of bioactive substances, especially in early spring.

## 1. Introduction

Common garlic (*Allium sativum* L.) is one of the oldest useful plants that have been known to man for thousands of years. Currently, it is cultivated in most countries located in a temperate zone [1]. The most frequently consumed part is the bulb. However, in Asian countries, the Balkans and the Podkarpacie region of Poland, the leaves are also used for this purpose [2,3]. In Asian and North American countries, the stems and young inflorescence shoots are also eaten [4].

The garlic bulb’s composition is about 60% water, 32% carbohydrates and 6.45% protein per fresh sample. The caloric content of garlic is quite high, amounting to 146 kcal/100 g fresh mass (FM) of the product. Numerous minerals can be identified in the bulbs: potassium (400 g), phosphorus (153 g), magnesium (25 mg), sodium (17 g), and calcium (41 mg/100 g FM). In addition, selenium and germanium are also present in garlic, and their amount depends on the content of minerals present in the soil [5,6]. Garlic cloves are a rich source of vitamin C (approx. 31 mg/100 g FW) and provides small amounts of B vitamins, especially vitamin B_1_ [7,8,9,10].

Garlic leaves have a 13–18% dry weight contain 4.6–6.6% sugars, and are rich in vitamin C (16–20 mg/100 g FW), fibre (1.3–2.2%) and chlorophyll (1–1.6 mg/kg FW) [8,11]. Their composition is comparable to other vegetables, such as savoy cabbage, rutabaga, brussels sprouts, kale or white cabbage [3]. Some studies indicate that the content of allicin in leaves is at a similar level to or higher than that of bulbs [2,12].

Garlic is valued for its bactericidal, antiparasitic, antiviral and antifungal properties. Compounds present in garlic have a beneficial effect on the human organism, mainly by the reduction in oxidative stress, which strengthens the immune system response to various diseases [13,14,15]. Numerous studies show the anti-cancer effect of garlic and its positive effect on the regulation of blood pressure, as well as maintaining the proper level of cholesterol and lipids in the blood [16,17,18]. Additionally, garlic strengthens and regulates the cellular responses of the immune system [14,19]. Due to its composition, especially with the content of allicin and other sulphur compounds, it can be referred to as a natural antibiotic [13,20]. Garlic compounds, according to some studies, affect the metabolism of adipose tissue, causing weight loss, and may also reduce the expression of the mRNA gene involved in adipogenesis [21,22]. The bioactive compounds of garlic and its products can lower blood glucose levels and affect insulin secretion [23,24]. Moreover, epidemiological and experimental studies show a relationship between garlic consumption and a reduced risk of oesophageal, brain, skin, stomach, liver, prostate, breast, colon, bladder and lung cancer [25,26,27,28].

Garlic is propagated only vegetatively. For this purpose, only cloves or air bulbs planted in the autumn or spring are used. Unfortunately, the seed material is often infected with viruses, which results in low yields [29]. In autumn, due to their poor storage, cultivars are planted that shoot into inflorescences [30].

Due to its exceptional nutritional and health benefits as well as its unique aroma, garlic is widely used by food and other industries [2,5,31,32]. Also, people are more and more aware of the beneficial effects of the medicinal substances contained in garlic and have thus been using it more often, not only for medicinal purposes, but also treating it as the main ingredient of food seasoning. In recent years, there has been an increased demand for fresh vegetables, which are dietarily and pharmacologically valuable. In the spring period, i.e., from March to May, there are shortages of fresh local vegetables on the market. Therefore, the harvesting of young leaves and young not-yet-cloned bulbs of garlic could enrich the range of vegetables available in spring.

The aim of this research was to compare the proximate composition, as well as the antioxidant activity and selected polyphenolic compounds content in the leaves and cloves of the winter garlic cultivars Harnaś and Ornak harvested in May, June and July.

## 2. Results

It was shown that the dry matter content in the cloves was even twice as high as in the leaves. The average dry matter content of the cloves in the 3-year harvest was 26.3% (Table 1).The highest dry matter content was found in the cloves of both cultivars in the July harvest compared to other samples (Table 1), which was statistically significant. The average dry matter content in the leaves of young garlic plants was 14.30% (Table 1). In the leaves of young garlic plants, no statistically significant differences were found in the dry matter content in relation to the cultivar and harvest date (Table 1). 

The average protein content of the leaves was 14.56 g/100 g DM (dry matter), and for the cloves, it was 11.40 g/100 g DM (Table 1). The protein content in the leaves decreased with the age of the plant. Harnaś leaves harvested in May were characterised by the highest amount of protein compared to leaves of both examined cultivars collected in July (Table 1). The lowest content of protein was found in the leaves of the Ornak cultivar collected in July. No statistical differences were found in the content of this component in the cloves (Table 1).

Garlic leaves were a richer source of crude fat than cloves (Table 1). The average crude fat content in the leaves was 1.75 g/100 g DM, while in the cloves, this value was almost three times lower and amounted to 0.64 g/100 g DM. Both the leaves and cloves harvested in the youngest stage of development, in May, contained significantly more crude fat compared to the samples collected in July.

The average ash content in the leaves in the tested material was 8.60 g/100 g DM (Table 1). The average ash content in the cloves of young garlic plants harvested for 3 years was 3.39 g/100 g DM (Table 1). The highest ash content was determined in the cloves of both cultivars collected in May compared to the samples collected in July. In the leaves of young garlic plants, neither the cultivar nor the harvest date had any influence on the ash content.

In the leaves of young garlic plants, the average total carbohydrate content was 75.07 g/100 g DM (Table 1). The cloves of garlic plants collected in all terms contained significantly more carbohydrates than the leaves (Table 2).

The leaves were characterised by a much higher vitamin C content during the three-year harvest than the cloves of young garlic plants (Table 2). Depending on the harvest date, it was 3–4 times greater. The highest vitamin C content was observed both in the leaves and cloves harvested in the youngest stage of plant development, in May.

The average content of polyphenols in the harvested leaves, from over the 3 years, was 1563.07 g/100 g DM, and in cloves, it was 321.8 g/100 g DM (Table 2). A significantly higher content of polyphenols was found in the leaves of young plants of the Ornak cultivar harvested in May compared to the June harvest (Table 2). A lower amount of polyphenols was found in cloves of the Ornak cultivar from the July harvest compared to the amount in cloves of the Harnaś cultivar harvested in June.

**Figure 1:**
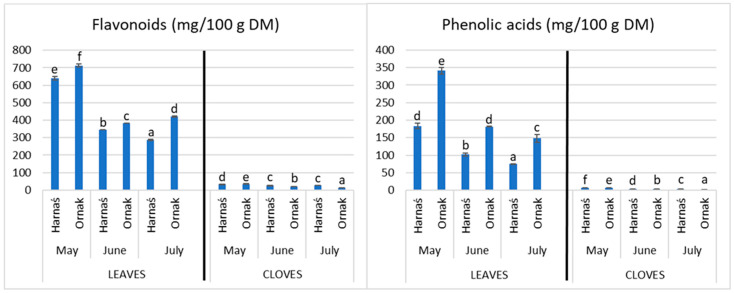
The sum of flavonoids and phenolic acids in the leaves and cloves of garlic Harnaś and Ornak cultivars harvested in various stages of development. Bars with different letters (a–f) are significantly different at *p* ≤ 0.05.

Antioxidant activity was determined in the leaves and cloves of young garlic plants using three methods: ABTS, DPPH and FRAP. Based on the results of above mentioned methods, garlic leaves, compared to cloves, are characterised by a 5–6-times higher antioxidant activity (Table 2). The highest antioxidant activity determined with ABTS and DPPH methods was measured in the leaves of the Ornak cultivar collected in June compared to the other leaves with the exception of Harnaś leaves collected in May. Only the FRAP method showed a significant impact of the May harvest date on the level of antioxidant activity, showing a higher level in garlic leaves and cloves compared to that in older plants. The content of the polyphenol profile was determined in the cloves and leaves of young garlic plants. Young garlic leaves were characterised by a higher content of flavonoids and phenolic acids compared to cloves. The Ornak cultivar, harvested in May, was revealed to have the highest significant total flavonoids content in garlic leaves, while the cultivar Harnaś harvested in July has the lowest (Figure 1). In the plant material from the cloves, the highest concentration of flavonoids was found in May in the Harnaś cultivar, and the lowest was found in the garlic of the Ornak cultivar in July. The highest content of phenolic acids was found in the leaves of young garlic plants of the Ornak cultivar harvested in May. The lowest concentration of these compounds was found in the July harvest in Harnaś cultivar (Figure 1).

The cloves of young garlic plants were characterised by a lower content of phenolic acids than the leaves with the exception of caffeic acids (Figure 1). The highest amount was found in the cloves of young plants from May of the Harnaś cultivar, and the significantly lowest concentration in the Ornak cultivar from July. For the analysis of individual polyphenolic compounds, the 17 compounds most characteristic of plant material were selected [21]. Ten flavonoids (Table 3) and seven phenolic acids (Table 4) were found in young garlic plants. The young garlic leaves of the Ornak cultivar harvested in May were characterised by the highest content of the analysed polyphenolic compounds. Among the analysed compounds, a significantly higher content of catechins was found (Table 3). In the material obtained from the garlic cloves, a significantly lower concentration of polyphenolic compounds was found compared to the material obtained from the leaves (Table 3 and Table 4). The highest significant content of the tested compounds was demonstrated in the May harvest of the cultivar Ornak, while the most dominant compound in the cloves of garlic was epicatechin (Table 3 and Table 4).

Using a screening test, two main factors with the highest factor loadings in relation to the component data (principal component (PC) 1 and 2) were determined. The analysis of the eigenvalues of the correlation matrix showed that the first and second principal components explained, respectively 83.88% and 91.5% of the total variance of the primary variables (Table 5).

Table 6 presents the factor coordinates of the analysed variables broken down into two main components. It was shown that PC 1 was highly correlated with all the variables defining the basic chemical composition (dry matter, proteins, crude fat, ash and total carbohydrates), the antioxidant activity (vitamin C, total polyphenols and DPPH), flavonoids and phenolic acids. It was also found that PC 2 was correlated with the month and proteins, with a value of 0.6.

**Figure 2:**
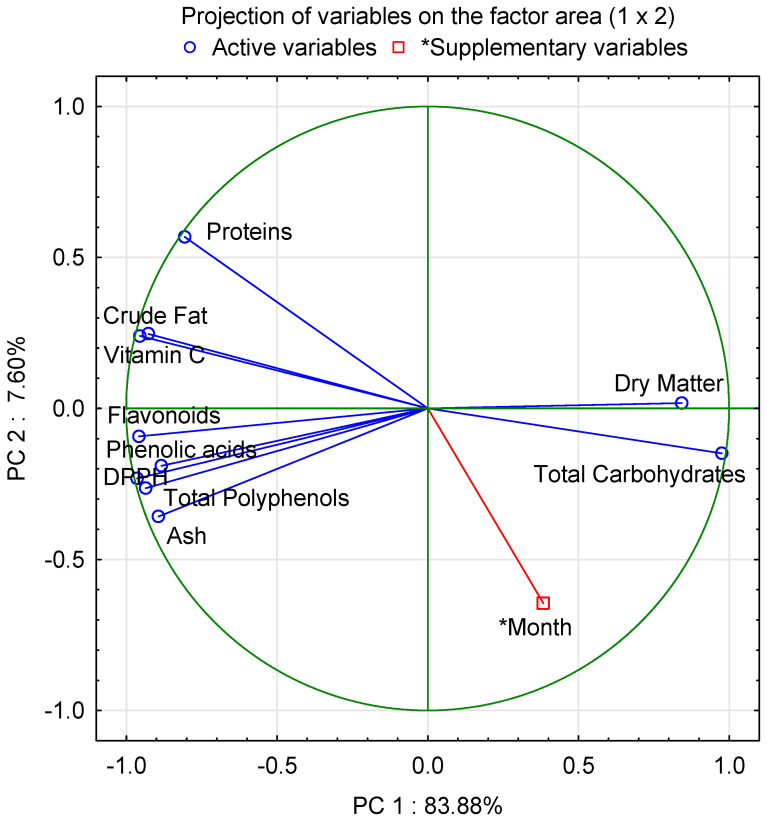
Projection of basic chemical composition (dry matter, proteins, crude fat, ash and total carbohydrates), the antioxidant activity (vitamin C, total polyphenols and DPPH), flavonoids and phenolic acids on the principal components area (1 × 2).

The relationships between the primary variables and PC 1 and PC 2 are presented graphically in Figure 2. A very strong positive correlation was found between dry matter and total carbohydrates (marked with a green ellipse), as well as a very strong positive correlation between crude fat, vitamin C, flavonoids, phenolic acids, DPPH, total polyphenols and ash (marked with a red ellipse). Both of these groups were very highly negatively correlated with each other, as evidenced by their arrangement on opposite sides of each other. Figure 2 also shows that the month and protein variables are located on opposite sides of the principal components area, which means that they are negatively correlated. The results are confirmed by the protein content because July differed from May and June due to the content of proteins (Table 6).

## 3. Discussion

Our research showed significant statistical differences between the cloves and leaves of young garlic plants in the content of dry matter, crude fat, vitamin C and antioxidant activity, which were determined using the FRAP method, as well as in the content of polyphenols. The authors, to the best of their knowledge, have only found limited information about the composition of garlic plants (leaves and cloves) harvested in various growing stages. The authors of published articles mainly focused on comparing the mature morphological parts of garlic, especially cloves [2,8,11,33].

The highest dry matter content was found in the cloves of young garlic plants compared to the leaves. The highest content was determined in the July harvest, but the cultivar did not affect the amount of dry matter at any clove harvesting dates. In the leaves of young plants, no effect of the harvest date or cultivar on the dry matter content was demonstrated. It was reported that various factors, including the selection of material for growing, the quality and moisture of the soil, climate condition and the stage of development during the harvest, can affect the proximate composition and bioactive compounds content in cloves of garlic [10]. Our results from the last harvesting time are similar to data published by Ciuba et al. [8]. These authors reported that the cloves of garlic from a different country (China, Spain and Poland) had a dry matter content in the range of 28.13–38.49 g/100 g fresh weight. They also reported that the content of dry matter in the mature cloves of cultivars Harnaś and Ornak were 38.49 g/100 g FM; and 37.70 g/100 g FM. Also Marciniec and Włodarczyk-Marciniec [34] reported that the dry matter content in garlic cloves was 37.00 g/100 g. Boonpeng et al. [35] showed a dry matter content in garlic from the northern part of Thailand in the amount of 35.63 g/100 g, whereas Suleria et al. [36] showed a dry matter content of 46.40 g/100 g. A higher content of DM (44%) was found in the cloves of the mature garlic of Polish origin was reported in studies by Jędrszczyk et al. [11]. The content of dry matter in leaves, in our study, was similar to the level reported by Piątkowska et al. [12]. These authors determined the dry matter of the leaves of mature garlic plants to be 11.89–12.25 g/100 g fresh weight. Jędrszczyk et al. [11] reported that the DM in mature leaves was 13.1 g/100 g FM. Similar results were published by Dyduch and Najda [3]. These authors reported the dry matter content in garlic leaves in the range of 10.7 and 15.8%.

The average protein content in cloves and leaves, in our research, was 11.41 g/100 g DM, and 14.56 g/100 g DM, respectively. The highest protein content was found in garlic leaves harvested in May, but the cultivar had no effect on the protein content. The harvest date and the cultivar did not affect the protein content in the cloves of garlic plants harvested at various time of growing. A higher content of protein in the leaves of garlic was reported by Piątkowska et al. [12]. These authors showed that the leaves of mature garlic plants of Polish-origin cultivars contained proteins in the range of 19.17–23.55 g/100 g DM. The available scientific literature shows the protein content in ripe garlic cloves in various ranges (DM) [11,34]. Marciniec and Włodarczyk-Marciniec [34] reported a higher content of protein in cloves (6.40 g/100 g FM, which is 17.00 g/100 g DM). Yasful et al. [37] reported the content of crude protein in the amount of 15.33 g/100 DM in cloves of garlic from Nigeria. Also Haciseferoĝullari et al. [32] showed a higher protein content in the cloves of garlic cultivated in Northern Turkey, reporting 9.26 g/100 g FM, i.e., 27.5 g/100 g DM. The difference in the content of crude protein in mature garlic cloves, between our results and the above-mentioned authors, can be explained by the various climate condition and soil quality during the growing of plants.

Young garlic leaves harvested in May and July had a higher crude fat content. The cultivar did not affect the content of the analysed component in the leaves and cloves of young plants. Research by Piątkowska et al. [12] showed a fat content between 1.34 g and 4.7 g/100 g DM in mature leaves. The crude fat content in mature garlic cloves of the Harnaś cultivar in the studies of Ciuba et al. [8] was 0.16 g/100 g FM (0.42 g/100 DM). Suleria et al. [36], based on USDA data, showed that the content of crude fat in garlic cloves was 5.10 g/100 g FM (10.99 g/100 g DM).

The leaves of young plants had a higher ash content than the cloves. However, the harvest date and cultivar had no effect on the content of this component. In the currently available scientific literature, the content of ash measured in garlic depends on the plant material’s country of origin. In studies conducted by Ciuba et al. [8], the ash content in Polish varieties of garlic cloves was found to be between 0.98 and 1.49 g/100 g DM, and contained 2.13 g/100 g FM (6.33 g/100 g DM) of ash. Suleria et al. [25] showed that the content of ash was 2.30 g/100 g FM (4.95 g/100 g DM). The ash content in garlic leaves was determined by Piątkowska et al. [12] to range between 11.02 g and 11.86 g/100 g DM). The ash content in the cloves analysed during our research showed a much lower content. This difference is due to the fact that our analyses were performed with the use of young garlic cloves, and that the above-mentioned studies concerned mature garlic cloves.

The content of total carbohydrates in garlic leaves and cloves differed from the results of other authors [8,34,36]. Research by Ciuba et al. [8] showed lower values in the range of 27.43 g and 30.52 g/100 g DM of total carbohydrates in the cloves of ripe garlic. Suleria et al. [36] showed also the lower content of this component at 41.40 g/100 g DM; Marciniec and Włodarczyk-Marciniec [34] found 28.60 g/100 g DM. Total carbohydrates in leaves analysed by Piątkowska et al. [12] were between 66.95 g and 74.85 g/100 g DM). These results are similar to our finding. Our own research showed no effect of the cultivar or the harvest date on the total carbohydrate content in the leaves or cloves of young garlic plants.

The young garlic leaves harvested in May were characterised by a higher vitamin C content, but the cultivar had no effect on the concentration of the analysed ingredient. Authors of other studies obtained significantly lower vitamin C values in mature garlic cloves compared to our results of the content of vitamin C in cloves harvested in May and June [11,38]. While the results of content of vitamin C in cloves harvested in July are in the same range as other authors [11,34], Ciuba et al. [8] obtained a lower content of vitamin C in mature cloves, between 11.08 mg and 9.53 mg/100 g DM. Research conducted by Piątkowska et al. [12] showed a vitamin C content in garlic leaves between 74.84 mg and 389.5 mg/100 g DM.

The leaves of young garlic plants harvested in May were distinguished by a higher total polyphenols content. There were no differences in the content of polyphenols between the cultivars, but we have found the difference in the content of phenolic compounds between the leaves and cloves. In studies available in the literature, significantly lower values of polyphenols in mature garlic leaves were observed [11,12]. Piątkowska et al. [9,12] showed that the content of polyphenols in mature garlic leaves was between 253.06 mg and 546.24 mg/100 g DM. In our research, the average content of polyphenols in the cloves of young garlic plants was 321.8 g/100 g DM. The highest content of polyphenols in the cloves of young garlic cultivar Ornak plants was observed in the May harvest. In our study, the samples of garlic cloves collected in July had a lower content of polyphenolic compounds compared to other authors [8,39]. Research by Ciuba et al. [8] showed the presence of polyphenols in ripe cloves of garlic, between 369.49 mg and 495.1 mg/100 g DM. According to Leelarungrayub et al. [40], the content of polyphenols was at the level of 450.0 mg/100 g DM and according to Mnayer et al. [39], in garlic, 561.0 mg/100 g DM was found. Research by Querioz et al. [41] showed the presence of polyphenols in garlic cloves in the amount of 699.0 mg/100 g DM.

In this study it was shown that there is a higher antioxidant activity in the leaves of young garlic plants than in the cloves. The cultivar had no effect on the antioxidant activity in leaves of young garlic plants. The analysis using the FRAP method showed the highest antioxidant activity of the May harvest. According to Piątkowska et al. [12] the antioxidant activity of the ABTS method in mature garlic leaves was in the range of 203.5 μmol and 230.6 μmol Trolox/1 g DM. The average antioxidant activity determined by the ABTS^+^ method in the cloves of young garlic plants was 61.86 μmol Trolox g/1 g DM. The highest activity was demonstrated in the May 2020 harvest of the Harnaś cultivar. Research by Ciuba et al. [8] show that the antioxidant activity in mature garlic cloves to be between 24.96 μmol and 25.28 μmol Trolox/1 g DM.

The leaves of young garlic plants were characterised by a higher content of flavonoids and phenolic acids. The highest concentration of flavonoids was found in the May harvest in garlic of the Ornak cultivar. Their content was influenced by the cultivar and date of harvest. On the other hand, the harvest date influenced the concentration of phenolic acids. Their highest content was found in garlic leaves of the Ornak cultivar from May. The highest level of phenolic compounds being found in young plants can be explained by their protective effect for growing plants. These compounds protect the plant against high or low ambient temperature, drought, and also pathogenic bacteria, fungi and viruses [42]. In the available literature, studies on the content of polyphenolic compounds were carried out on ripe garlic cloves. To the best of the authors’ knowledge, there are no studies on young garlic plants divided into morphological parts, i.e., leaves and cloves. Kim et al. [43] showed the presence of three polyphenolic compounds in mature garlic cloves: caffeic acid, 0.748 mg/100 g DM; p-coumaric acid, 0.125 mg/100 g DM; and ferulic acid, 0.157 mg/100 g DM. On the other hand, the total content of polyphenolic compounds was shown at the level of 1.786 mg/100 g DM. In a study by Miean and Mohamed [44] in garlic cloves, the amount of myricetin was found to be at 6.9 mg/100 g DM and that of quercetin at 4.7 mg/100 g DM was demonstrated. In these studies, the content of luteolin and kaempferol was also determined; however, the presence of these compounds was not demonstrated in the tested garlic. However, in the studies by Gorinstein et al. [45], the following polyphenolic compounds were determined in cloves of ripe garlic: vanillic acid (0.27 mg/100 g DM), caffeic acid (0.86 mg/100 g DM), coumarin (0.02 mg/100 g DM), ferulic acid (0.03 mg/100 g DM), sinapic acid (0.05 mg/100 g DM), quercetin (8.06 mg/100 g DM), and kaempferol (0.10 mg/100 g DM). In our research, the content of polyphenolic compounds was much higher than in the above-cited publications. This is due to the fact that the authors of the above studies conducted research on mature garlic cloves, while our research was carried out on young cloves and leaves harvested in early spring, in which the presence of polyphenols and individual polyphenolic compounds is much higher than in fully mature plants. The content of polyphenols in a sample also depends on other factors, including climatic conditions and the time of analysis after harvesting the plant, because some of the water may evaporate naturally and change the proportions of the compounds. Storage conditions, e.g., inappropriate temperature and high humidity, may affect the increased synthesis of polyphenols [42]. Important factors that may affect the result of the analysis of polyphenolic profile and content will be the method of extract storage (temperature, access to light and air) [46,47]. The most stable compounds in the group of polyphenols are phenolic acids, and the least stable are anthocyanins, where especially the presence of oxygen and a temperature of about 40 °C can significantly affect their content [47,48,49,50].

In this study we have found that the content of phenolic compounds, vitamin C and antioxidant activity measured with ABTS, DPPH and FRAP methods had higher value in the same samples of plant material (Table 2, Table 3 and Table 4; Figure 1). The higher values were found using the ABTS and FRAP methods, especially in leaves. It is well know that in ABTS method, the hydrophilic and hydrophobic antioxidants react with ABTS radicals. It was also reported that some phenolic compounds, for example, flavonoids and simple polyphenols, react with ABTS^•+^, and that coumaric acid, for example, has weak this radical scavenging activity [51,52,53]. The lowest values were measured using the DPPH method. This can be explained by the fact that DPPH is soluble only in organic solvents and only reacts with hydrophobic substances. It was also reported that substituents of the phenolic ring strongly affect the possibility of scavenging DPPH radicals [51,53].

## 4. Materials and Methods

### 4.1. Plant Material

The experiment was conducted in 2018–2020 at the Experimental Station of the Department of Horticulture, University of Agriculture, Hugo Kołłątaj in Krakow. The research material consisted of leaves and cloves of two winter garlic cultivars: Harnaś and Ornak.

The cloves intended for planting were purchased from Krakowska Hodowla Nasion Polan, Kraków, Poland. Garlic was planted in brown soil every year after October 25 at a distance of 30 cm between the rows and 10 cm in a row, and at a depth of 5–6 cm. In Poland, garlic is planted around October 25 so as to be in time before the ground freezes completely. Garlic planted at this time will have time to root well, but it will not grow leaves before winter unless it is still warm. Garlic planted in autumn is harvested in July. During the experiment, maintenance treatments typical for the species were carried out: weeding, regularly as needed; and irrigation, regularly in dry weather. Harvesting was performed on three dates: in May, June and July. This occurred for three years, always at the same stage of development. In May and June, unripe plants were harvested, that is, young leaves and bulbs that had not yet been divided, and in July, plants were harvested at their full maturity stage. The plants were cleaned and next prepared for freeze drying.

### 4.2. Proximate Analysis of Plant Material

The dry matter content was determined in fresh material. This method consisted of determining the loss of weight after removing water from the product during thermal drying at 105 °C under normal pressure. The determination was performed in two parallel replications, and the final result was the arithmetic mean. A Memmert GmbK laboratory dryer (Schwabach, Germany) was used to perform this dry matter determination. The remaining plant material was freeze-dried with a Christ Alpa 1–4 laboratory freeze dryer (Gefriertrocknungsanlangen, Osterode am Harz, Germany).

The proximate analysis of freeze-dried samples of garlic was measured according to the AOAC official methods [54]. The concentration of protein was measured with the Kjeldahl method (AOAC no. 978.04), crude fat content in accordance with the Soxhlet method (AOAC no. 935.38) and ash (AOAC no. 930.05). Dry matter was determined using an laboratory oven (Memmert GmbK, Schwabach, Germany). For the nitrogen content, the Kiejdalh method was used with equipment designed for the digestion and distillation of samples (FOSS Digester and Autodistillation Unit Kjeltec^TM^ 8200; Tecator Foss, Hillerød, Sweden). About 0. 500 g of sample was weighed on an analytical scale (accuracy 0.0001) and transferred to the mineralisation tube with catalysators, e.g., CuSO_4_ and K_2_SO_4_ (POCh, Katowice, Poland). Next, 14 cm^3^ of sulphuric acid was added to the sample. Mineralisation was performed at 420 °C in the mineralisation unit. The mineralisation was performed to obtain a light-green-coloured sample. The mineralisation tube was then cooled After cooling, the flask with the mineraliser was moved into the Autodistillation Unit Kjeltec^TM^ 8200 for distillation. The obtained distillate was titrated with 0.1 M HCl solution (POCh, Katowice, Poland) until a light purple colour was formed. The percentage of total nitrogen was calculated according to formula N = (V × M × 14.007 × 100)/m, where N is the total nitrogen content in 100 g of the test material (g/100 g); V is the volume of HCl used for titration of the sample (cm^3^); M is the molar concentration of HCl (mol/dm^3^); 14.007 is the amount of nitrogen, which corresponds to 1 cm^3^ of HCl with a concentration of 1 mol/dm^3^; and m is the sample mass [g]. The factor 6.25 was used to calculate the content of protein in the garlic samples.

The crude fat content was determined using the Soxhlet method, and a Soxtec Avanti’s 2050 Auto Extraction Unit was used (Tecator Foss, Hillerød, Sweden). About 1 g of the tested sample was weighed with an accuracy of 0.0001 g and placed in the extraction thimble. The clean vessel was dried in an incubator at 105 °C, cooled in a desiccator, and weighed to the nearest 0.0001 g. The vessel was filled with petroleum ether (POCh, Katowice, Poland) in a volume of 80 cm^3^. Thimbles with sample weights and vessels with ether were placed in the Soxhlet apparatus for a specified time (about 1.5 h, with 20 min for heating, 40 min for extraction, 15 min for the cooling down of sample), with fat extraction being automatically performed. After the analysis was completed, the vials with fat were placed in a dryer at a temperature of 105 °C for 15 min, then transferred to a desiccator to cool down. Dried and cooled vials with crude fat were weighed on an analytical scale with an accuracy of 0.0001 g. The crude fat content was calculated according to the formula:

Crude fat content (g/100 g) = [(b − a) × 100]/m, where a is the weight of an empty vial before analysis (g), b is the weight of the vial with crude fat after analysis (g), and mis the sample mass (g).

The ash content was measured in the electric muffle furnace (SNOL 82/110, Utena, Lithuania).

The total carbohydrates content in dry matter was calculated based on the following formula: total carbohydrates = 100 – (protein + raw fat + ash) [55]. In the methanolic extract received from freeze dry samples, the total phenolic content and antioxidant activity was measured.

### 4.3. Vitamin C, Total Phenolic Compounds Content and Antioxidant Activity

The content of vitamin C (as the sum of ascorbic and dehydroascorbic acid) in the fresh plant material was determined using the Thillmans method following the modifications made by Pijanowski [56]. In this method, dehydroascorbic acid is reduced to ascorbic acid in the presence of sodium sulphide. The excess amount of sodium sulphide is precipitated by mercury chloride solution. The sum of ascorbic acid and dehydroascorbic acid is determined by titration with 2,6-dichlorophenolindophenol.

Methanol extracts were prepared from fresh material. In order to prepare them, 5 g of fresh sample and 80 mL of 70% methanol (POCh, Katowice, Poland) were used. The samples were extracted for 2 h at room temperature and protected from light. Then, the samples were filtered. After filtration, the extracts were stored at −20 °C. They were used for the future analysis of total polyphenols and antioxidant activity.

The total polyphenols content was measured spectrometrically using Folin-Ciocalteau reagent, at a wavelength of 760 nm using a RayLeigh UV-1800 spectrophotometer (UV-1800 spectrophotometer, Beijing Beifen-Ruili Analytical Instrument Co., Ltd., Beijing, China) [57]. The results are expressed as the chlorogenic acid equivalent (CGA) in mg per 100 g of dry matter (DM).

The antioxidant activity of methanolic extracts of garlic samples was measured using the following methods: with ABTS^•+^ radical (2,2′-azino-bis-(3-ethylbenzothiazoline-6-sulfonic acid); Sigma Aldrich, Saint Louis, MO, USA) [58]; DPPH (2,2-diphenyl-1-picrylhydrazyl, Sigma Aldrich, Saint Louis, MO, USA) [59]; and the FRAP method (ferric-reducing antioxidant power) [60], as was previously reported [61].

The working solution for ABTS analysis was received by diluting the ABTS^•+^ stock solution with 70% methanol to receive an absorbance between 0.740 and 0.750 at 734 nm. A total of 10–100 μL of the extract of garlic was transferred into a test tube and diluted up to 1 mL with 70% methanol. After the mixing of a diluted extract with 2 mL of the working solution of the ABTS^•+^, the mixture was kept in dark at 30 °C for 6 min. The absorbance of the sample was measured at a wavelength of 737 nm.

The working solution of DPPH^•^ was prepared by dissolving 6 mg of DPPH in 100 mL of methanol. The working solution was obtained by diluting the stock solution with methanol to obtain an absorbance of 0.900–1.000 at 515 nm. A total of 10–200 μL of the extract was put into a test tube and diluted up to 1.5 mL with methanol. After mixing the diluted extract with 3 mL of the DPPH^•^ solution, the mixture was kept in the dark at room temperature for 10 min. The absorbance of the sample was measured at a wavelength of 515 nm.

The total reducing capability, using the FRAP method, was determined as reported by Benzie and Strain [60]. The extracts (10–200 μL) were put into a test tube and diluted up to 1 mL with 70% methanol. After mixing the diluted extract with 3 mL of the working solution of the FRAP reagent, the mixture was kept in the dark at room temperature for 10 min. The absorbance of the samples was measured at 593 nm.

The results obtained for ABTS^●+^, DPPH and FRAP methods were compared to the concentration–response curve of the standard Trolox dilution, and the results obtained are expressed in μM Trolox/1 g DM of sample.

### 4.4. HPLC Analysis of Plant Material

For the HPLC polyphenolic compounds, an acidified methanolic extract of garlic was used. One g of the lyophilised sample was taken and extracted using a laboratory shaker Elpin Plus, type 357 (Lubawa, Poland) for 2 h at room temperature while protected from light in 40 mL of 70% methanol (POCh, Katowice, Poland) and acidified with formic acid (0.1%) (Chempur, Piekary Śląskie, Poland). The samples were then filtered, and the extracts were stored at −20 °C [61,62]. Methanolic extracts were used to determine polyphenolic compounds via the HPLC method using the Prominence-i LC 2030C D3 Plus system (Shimandzu, Kyoto, Japan) along with a DAD detector and a Luna Omega 5 µm Polar C18, 100A, 250 × 10 mm Phenomenex column (California, USA). The separation was performed at 40 °C. The mobile phase was a mixture of two eluents: A, 0.1% formic acid in water (*v*/*v*); and B, 0.1% formic acid in methanol (*v*/*v*). The flow rate of the mobile phase was 1.2 mL/min. The analysis was carried out with the following gradient conditions: from 20% to 40% B in 10 min, 40% B for 10 min, from 40% to 50% B in 10 min, from 50% to 60% B in 5 min, 60% B for 5 min, from 60 to 70% B in 5 min, from 70% to 90% B in 5 min, 90% B for 5 min, from 90% to 20% B (the initial condition) in 1 min, and 20% B for 4 min. This resulted in a total run time of 60 min. The injection volume was 20 μL. A stock standard solution (100 mg/L) of each polyphenolic compound was prepared in 0.1% formic acid in 70% methanol (*v*/*v*) (POCH, Poland). The calibration curves of the polyphenol standards were made by the dilution of stock standard solutions in 0.1% formic acid in 70% methanol (*v*/*v*). All the solutions were filtered through a 0.22 µm filter.

The following polyphenolic compounds were determined based on standards, namely catechin, epicatechin, naringin, hesperidin, myricetin, quercetin, luteolin, kaempferol, apigenin, rutin, chlorogenic acid, 4-hydroxybenzoic acid, caffeic acid, vanillic acid, p-coumaric acid, ferulic acid, and sinapic acid (Sigma Saint Louis, MO, USA), based on LabSolution ver. 5.93 Shimandzu Corporation (Kyoto, Japan), who determined their concentration in the plant material, as was reported by Sadowska et al. [63]. The content of quercetin, luteolin, apigenin, and myricetin were measured only in their free aglycones form.

### 4.5. Statistical Analysis

The analyses were performed in two or three parallel replications. Results are reported as the means ± SD. One and two way factorial analysis of variance (ANOVA) was used to test the differences. The significance of the obtained differences were verified using Duncan’s test at the level of significance of *p* ≤ 0.05.

In this study, Principal Component Analysis (PCA) was used to identify the factors that were common to the primary variables, defining the basic chemical composition (dry matter, proteins, crude fat, ash and total carbohydrates), the antioxidant activity (vitamin C, total polyphenols and DPPH), flavonoids and phenolic acids, and for the month of garlic harvest. By reducing the number of primary variables and replacing them with components that significantly explain their variability, PCA analysis makes it possible to describe the ongoing processes and phenomena with the maximum amount of information [64]. The screening test proposed by Cattell [65] was used to determine the number of principal components. The last step was a graphical presentation of the dataset, where each variable was represented by a vector, and its direction and length determined the degree to which individual variables influenced the principal components. In the case of the location of the analysed variables near the circumference of the circle, the majority of the information contained in them is carried by the main components. A strong positive correlation occurs when the variables are located next to each other, while the vectors of the variables located perpendicular to each other indicate its absence. Placing the variables on opposite sides of each other means that they are negatively correlated.

The results were subjected to analysis with the use of STATISTICA v. 13.3 (StatSoft Inc., Tulsa, OK, USA).

## 5. Conclusions

It was found that the time of harvest had a significant effect on the proximate composition, especially of leaves. The leaves, harvested in May, were a better source of protein, crude fat and vitamin C than the leaves of a fully developed plant (harvested in July). Only the level of total carbohydrates increased in the leaves as the plant developed. Less variability of the proximate composition during development was found in the cloves. Young cloves harvested in May, compared to those fully developed (harvest in July), only contained more protein, fat and ash. In the tested garlic cloves and leaves, 17 polyphenolic compounds were determined. The leaves had statistically higher amounts of all determined polyphenols, with the exception of epicatechin, coffee acid (less than in cloves) and myricetin (no differences). Based on the research, it was found that the leaves are a comparable or higher source of active ingredients than the cloves. Young garlic plants harvested in May are a valuable source of both nutrients and non-nutrients compounds.

## Figures and Tables

**Table 1:** The proximate analysis of the morphological parts of garlic obtained from Harnaś and Ornak cultivars harvested in various stages of development.

Cultivar and Time of Harvest #	Dry matter **	Proteins ***	Crude Fat ***	Ash ***	Total Carbohydrates ***
HL * May	13.65 ± 0.45 ^a^	19.86 ± 0.13 ^c^	2.45 ± 0.13 ^d^	8.30 ± 0.10 ^de^	69.38 ± 0.20 ^a^
HC * May	18.50 ± 0.74 ^a^	12.33 ± 0.35 ^abc^	1.23 ± 0.02 ^c^	5.18 ± 0.08 ^bcd^	81.26 ± 0.37 ^cd^
OL * May	14.23 ± 1.10 ^a^	15.21 ± 0.10 ^c^	2.43 ± 0.28 ^d^	9.18 ± 0.22 ^d^	73.17 ± 0.43 ^ab^
OC * May	16.81 ± 0.52 ^a^	14.03 ± 0.31 ^abc^	0.83 ± 0.03 ^abc^	5.08 ± 0.06 ^bcd^	80.06 ± 0.32 ^cd^
HL * June	14.44 ± 0.53 ^a^	14.38 ± 0.14 ^bc^	2.14 ± 0.25 ^d^	7.17 ± 0.13 ^bcde^	76.31 ± 0.14 ^bc^
HC * June	23.98 ± 1.23 ^b^	10.45 ± 0.15 ^a^	0.65 ± 0.21 ^abc^	4.21 ± 0.15 ^abc^	84.69 ± 0.36 ^d^
OL * June	15.34 ± 0.33 ^a^	13.29 ± 0.12 ^abc^	1.26 ± 0.13 ^c^	7.31 ± 0.28 ^cde^	78.13 ± 0.19 ^bc^
OC * June	25.08 ± 0.93 ^b^	10.74 ± 0.13 ^ab^	0.52 ± 0.05 ^ab^	3.70 ± 0.10 ^ab^	85.04 ± 0.13 ^d^
HL * July	12.77 ± 0.50 ^a^	13.79 ± 0.21 ^abc^	1.16 ± 0.12 ^bc^	9.46 ± 0.15 ^d^	75.59 ± 0.30 ^bc^
HC * July	34.84 ± 0.85 ^c^	10.19 ± 0.13 ^a^	0.29 ± 0.03 ^a^	3.13 ± 0.10 ^a^	86.39 ± 0.16 ^d^
OL * July	15.39 ± 0.30 ^a^	10.82 ± 0.13 ^ab^	1.06 ± 0.03 ^bc^	10.26 ± 0.16 ^d^	77.85 ± 0.15 ^bc^
OC * July	34.80 ± 0.80 ^c^	10.68 ± 0.27 ^ab^	0.32 ± 0.04 ^a^	2.38 ± 0.06 ^a^	86.61 ± 0.29 ^d^
L average ##	14.30 ± 0.53 A	14.56 ± 0.14 B	1.75 ± 0.16 B	8.60 ± 0.17 B	75.07 ± 0.24 A
C average ###	26.30 ± 0.84 B	11.40 ± 0.22 A	0.64 ± 0.06 A	3.39 ± 0.09 A	84.01 ± 0.27 B

# All results are the average from 3 years of sample collection; ## average from 3 years for leaves; ### average from 3 years for cloves * HL, Harnaś leaves; HC, Harnaś cloves; OL, Ornak leaves; OC, Ornak cloves; Values in columns with different letters (^a–e^) are significantly different at *p* ≤ 0.05. ** g/100 g FM (fresh matter); values with capital letter (A,B) within two last rows are statistically different at *p* ≤ 0.05 for morphological parts *** g/100 g DM (dry matter).

**Table 2:** Vitamin C (mg/100 g DM), total polyphenols (mg chlorogenic acid per 100 g DM) content and antioxidant activity (µmol Trolox/1 g DM) of morphological parts of garlic from Harnaś and Ornak cultivar harvested in various stages of development over 3 years.

Cultivar and Time of Harvest #	Vitamin C	Total Polyphenols	ABTS	DPPH	FRAP
HL * May	736.14 ± 25.51 ^c^	1344.73 ± 41.61 ^bc^	170.78 ± 5.74 ^cd^	31.26 ± 0.93 ^bc^	101.40 ± 3.05 ^c^
HC * May	239.38 ± 11.11 ^ab^	373.08 ± 8.52 ^a^	35.74 ± 1.73 ^a^	6.46 ± 0.23 ^a^	23.30 ± 0.24 ^a^
OL * May	732.55 ± 59.72 ^c^	1581.87 ± 103.09 ^c^	204.44 ± 14.09 ^d^	38.3 ± 1.99 ^c^	115.09 ± 7.53 ^c^
OC * May	216.09 ± 12.47 ^ab^	385.77 ± 7.45 ^a^	39.42 ± 1.65 ^a^	6.67 ± 0.11 ^a^	20.99 ± 0.70 ^a^
HL * June	426.71 ± 1.14 ^b^	1513.50 ± 37.93 ^c^	123.05 ± 2.20 ^b^	22.44 ± 0.43 ^b^	72.15 ± 1.80 ^b^
HC * June	95.29 ± 3.82 ^a^	419.24 ± 13.58 ^a^	43.75 ± 1.60 ^a^	4.53 ± 0.15 ^a^	16.98 ± 0.74 ^a^
OL * June	402.61 ± 8.04 ^b^	1095.63 ± 13.53 ^b^	129.07 ± 2.96 ^bc^	23.95 ± 0.39 ^b^	70.54 ± 0.68 ^b^
OC * June	95.29 ± 2.30 ^a^	303.54 ± 16.84 ^a^	22.58 ± 0.96 ^a^	3.36 ± 0.06 ^a^	9.29 ± 0.21 ^a^
HL * July	434.18 ± 23.72 ^b^	1345.12 ± 50.43 ^bc^	150.95 ± 5.77 ^bc^	26.89 ± 1.08 ^b^	75.37 ± 2.31 ^b^
HC * July	31.40 ± 3.65 ^a^	251.28 ± 1.87 ^a^	27.97 ± 0.40 ^a^	4.02 ± 0.04 ^a^	10.41 ± 0.15 ^a^
OL * July	201.38 ± 9.10 ^ab^	1401.95 ± 22.04 ^bc^	141.84 ± 2.13 ^bc^	28.77 ± 0.19 ^bc^	78.13 ± 0.81 ^b^
OC * July	23.60 ± 4.22 ^a^	197.89 ± 4.45 ^a^	19.86 ± 0.36 ^a^	3.07 ± 0.02 ^a^	7.59 ± 0.10 ^a^
L average ##	488.93 ± 2.20 B	156.07 ± 44.70 B	153.35 ± 4.74 B	28.60 ± 0.83 B	85.45 ± 2.70 B
C average ###	116.84 ± 6.26 A	321.8 ± 8.78 A	31.55 ± 11 A	4.68 ± 0.10 A	14.76 ± 0.35 A

# All results are the average from 3 years of sample collection, ## average from 3 years for leaves; ### average from 3 years for cloves * HL, Harnaś leaves; HC, Harnaś cloves; OL, Ornak leaves; OC, Ornak cloves; Values in columns with different letters (^a–d^) are significantly different at *p* ≤ 0.05; values with capital letter (A,B) within two last rows are statistically different at *p* ≤ 0.05 for morphological parts.

**Table 3:** The flavonoids content of morphological parts of garlic from Harnaś and Ornak cultivars harvested in various stages of development over 3 years (mg/100 g DM).

Cultivar and Time of Harvest #	Catechin	Epicatechnin	Naringin	Hesperidin	Myricetin	Quercetin	Luteolin	Kaempferol	Apigenin	Rutin
HL* May	491.2 ± 9.81 ^a^	94.26 ± 3.72 ^e^	7.36 ± 0.15 ^a^	9.94 ± 0.09 ^ab^	10.12 ± 0.06 ^bcd^	2.60 ± 0.00 ^cde^	2.04 ± 0.01 ^a^	1.97 ± 0.73 ^abcd^	2.42 ± 0.03 ^a^	17.42 ± 3.77 ^a^
HC * May	2.71 ± 0.19 ^a^	10.35 ± 0.60 ^a^	4.61 ± 0.17 ^a^	0.33 ± 0.22 ^a^	10.94 ± 0.05 ^bd^	ND	0.90 ± 0.02 ^abc^	1.56 ± 0.62 ^abcd^	1.98 ± 0.00 ^ab^	
OL * May	578.06 ± 4.51 ^c^	88.94 ± 2.92 ^de^	7.93 ± 0.20 ^ab^	7.01 ± 0.13 ^a^	10.53 ± 0.12 ^bd^	0.60 ± 0.00 ^abc^	0.93 ± 0.00 ^abc^	0.79 ± 0.71 ^ab^	2.43 ± 0.01 ^a^	6.49 ± 0.02 ^a^
OC * May	2.78 ± 0.39 ^b^	5.78 ± 0.01 ^a^	7.04 ± 0.01 ^a^	0.22 ± 0.00 ^a^	13.95 ± 0.08 ^d^	ND	1.00 ± 0.02 ^abc^	2.39 ± 0.01 ^cd^	2.32 ± 0.01 ^a^	ND
HL * June	204.22 ± 0.93 ^a^	82.97 ± 0.58 ^cde^	14.94 ± 0.10 ^b^	10.03 ± 0.02 ^ab^	9.30 ± 0.04 ^bd^	4.54 ± 0.02 ^de^	1.87 ± 0.01 ^bc^	2.35 ± 0.00 ^cd^	2.02 ± 0.00 ^a^	13.90 ± 0.95 ^a^
HC * June	2.37 ± 0.19 ^a^	8.06 ± 0.04 ^a^	2.55 ± 0.09 ^a^	0.83 ± 0.00 ^a^	8.68 ± 0.00 ^bcd^	0.41 ± 0.00 ^ab^	0.42 ± 0.01 ^a^	1.34 ± 0.01 ^abcd^	1.31 ± 0.01 ^abc^	ND
OL * June	285.62 ± 2.13 ^b^	54.28 ± 0.53 ^b^	9.33 ± 0.04 ^b^	5.88 ± 0.06 ^a^	8.14 ± 0.16 ^bc^	3.98 ± 0.02 ^de^	0.94 ± 0.01 ^abc^	1.40 ± 0.00 ^abcd^	1.95 ± 0.00 ^ab^	10.63 ± 1.07 ^a^
OC * June	1.58 ± 0.01 ^a^	5.59 ± 0.04 ^a^	3.06 ± 0.01 ^a^	ND	8.31 ± 0.03 ^bc^	ND	ND	0.88 ± 0.00 ^abc^	1.24 ± 0.00 ^abc^	ND
HL * July	72.50 ± 1.20 ^a^	49.08 ± 0.91 ^b^	22.17 ± 0.13 ^c^	47.28 ± 0.06 ^c^	9.68 ± 0.31 ^bd^	6.28 ± 0.06 ^e^	4.67 ± 0.07 ^d^	2.66 ± 0.01 ^d^	3.88 ± 0.03 ^d^	68.78 ± 0.53 ^c^
HC * July	4.01 ± 0.02 ^a^	10.02 ± 0.01 ^b^	3.76 ± 0.02 ^a^	0.77 ± 0.01 ^a^	4.79 ± 0.02 ^ab^	0.33 ± 0.00 ^ab^	0.66 ± 0.01 ^ac^	1.19 ± 0.00 ^abcd^	1.11 ± 0.00 ^bc^	0.33 ± 0.02 ^a^
OL *July	274.20 ± 1.12 ^b^	59.38 ± 0.34 ^bcd^	8.22 ± 0.14 ^a^	21.19 ± 2.70 ^b^	9.15 ± 0.05 ^bcd^	2.21 ± 0.03 ^bcd^	2.15 ± 0.03 ^b^	1.95 ± 0.15 ^bcd^	1.79 ± 0.03 ^abc^	41.12 ± 0.15 ^d^
OC * July	2.02 ± 0.01 ^a^	3.72 ± 0.01 ^a^	2.23 ± 0.01 ^a^	ND	3.08 ± 0.00 ^a^	ND	0.21 ± 0.00 ^a^	0.28 ± 0.00 ^ad^	0.75 ± 0.30 ^c^	ND
L average ##	317 ± 22 B	7.3 ± 2.0 A	11.6 ± 2.4 B	16.1 ± 1.5 B	9.5 ± 3.3 A	3.4 ± 1.3 B	2.1 ± 1.5 B	1.85 ± 1.2 B	2.41 ± 1.1 B	24.1 ± 11.8 B
C average ###	2.6 ± 0.3 A	71.5 ± 3.9 B	3.9 ± 0.8 A	0.35 ± 0.08 A	8.3 ± 5.6 A	0.13 ± 0.09 A	0.53 ± 0.2 A	1.27 ± 1.1 A	1.45 ± 0.8 A	3.02 ± 1.2 A

# All results are the average from 3 years of sample collection; ## average from 3 years for leaves; ### average from 3 years for cloves * HL, Harnaś leaves; HC, Harnaś cloves; OL, Ornak leaves; OC, Ornak cloves; values in columns with different letters (^a–e^) are significantly different. *p* ≤ 0.05; ND, not detected; values with capital letter (A,B) within two last rows are statistically different at *p* ≤ 0.05 for morphological parts.

**Table 4:** The phenolic acids content in morphological parts of garlic from Harnaś and Ornak cultivars harvested in various stages of development over 3 years (mg/100 g DM).

Cultivar and Time of Harvest #	Chlorogenic Acid	4-Hydroxybenzoic Acid	Coffee Acid	Vanillic Acid	p-coumaric Acid	Ferulic Acid	Sinapic Acid
HL * May	52.55 ± 0.07 ^cd^	44.21 ± 0.25 ^d^	2.52 ± 0.09 ^cd^	3.38 ± 0.19 ^b^	5.45 ± 2.38 ^b^	13.85 ± 0.08 ^d^	15.14 ± 0.34 ^b^
HC * May	1.35 ± 0.00 ^a^	1.34 ± 0.03 ^a^	0.87 ± 0.00 ^abc^	0.13 ± 0.00 ^a^	0.59 ± 0.00 ^a^	1.00 ± 0.00 ^a^	0.54 ± 0.00 ^a^
OL * May	106.87 ± 0.32 ^d^	91.48 ± 0.09 ^e^	3.67 ± 0.02 ^d^	6.48 ± 0.07 ^c^	10.60 ± 0.14 ^c^	12.56 ± 0.27 ^c^	23.39 ± 0.15 ^c^
OC * May	1.32 ± 0.01 ^a^	1.36 ± 0.01 ^a^	1.07 ± 0.01 ^abc^	0.11 ± 0.00 ^a^	1.20 ± 0.00 ^ab^	1.16 ± 0.01 ^a^	0.12 ± 0.00 ^a^
HL * June	29.05 ± 0.25 ^abc^	21.66 ± 4.99 ^abc^	1.80 ± 0.00 ^abc^	2.26 ± 0.04 ^b^	3.63 ± 0.05 ^ab^	6.66 ± 0.07 ^c^	16.98 ± 0.09 ^bc^
HC * June	0.69 ± 0.00 ^a^	0.76 ± 0.00 ^a^	0.46 ± 0.00 ^ab^	0.31 ± 0.00 ^a^	1.08 ± 0.00 ^ab^	0.62 ± 0.00 ^a^	0.41 ± 0.00 ^a^
OL * June	63.29 ± 0.01 ^jd^	40.79 ± 0.15 ^cd^	1.48 ± 0.01 ^abc^	3.25 ± 0.07 ^b^	4.83 ± 0.00 ^ab^	4.37 ± 0.06 ^bc^	15.58 ± 0.14 ^b^
OC * June	1.00 ± 0.01 ^a^	0.45 ± 0.00 ^a^	0.33 ± 0.00 ^ab^	ND	0.32 ± 0.00 ^a^	0.19 ± 0.00 ^a^	0.25 ± 0.00 ^a^
HL * July	13.33 ± 0.14 ^ab^	12.55 ± 0.10 ^ab^	2.23 ± 0.04 ^cd^	3.06 ± 0.26 ^b^	3.36 ± 0.04 ^a^	3.10 ± 0.14 ^ab^	13.96 ± 0.08 ^b^
HC *July	0.47 ± 0.00 ^a^	0.46 ± 0.01 ^ab^	0.45 ± 0.00 ^ab^	0.14 ± 0.00 ^a^	2.28 ± 0.00 ^ab^	0.42 ± 0.00 ^ca^	0.48 ± 0.00 ^a^
OL * July	32.49 ± 0.17 ^bc^	33.08 ± 1.60 ^bcd^	2.02 ± 0.00 ^a^	3.40 ± 0.04 ^ab^	2.95 ± 0.00 ^ab^	4.18 ± 0.00 ^bc^	20.23 ± 0.08 ^bc^
OC * July	0.21 ± 0.00 ^a^	0.35 ± 0.00 ^a^	0.16 ± 0.00 ^a^	0.32 ± 0.00 ^a^	0.37 ± 0.00 ^a^	0.25 ± 0.00 ^a^	0.10 ± 0.00 ^a^
L average ##	49.6 ± 42 B	40.6 ± 37.7 B	0.55 ± 0.4 A	3.64 ± 2.26 B	4.85 ± 2.21 B	7.45 ± 4.6 B	17.55 ± 8.3 B
C average ##	0.85 ± 0.52 A	0.78 ± 0.51 A	2.28 ± 1.77 B	0.17 ± 0.01 A	1.22 ± 0.8 A	0.60 ± 0.33 A	0.132 ± 0.01 A

# All results are the average from 3 years of sample collection, ## average from 3 years for leaves; ### average from 3 years for cloves * HL, Harnaś leaves; HC, Harnaś cloves; OL, Ornak leaves; OC, Ornak cloves; Values in columns with different letters (^a–e^) are significantly different. *p* ≤ 0.05; ND, not detected; values with capital letter (A,B) within two last rows are statistically different at *p* ≤ 0.05 for morphological parts for the three-year sample collections.

**Table 5:** Eigenvalues of the correlation matrix.

Principal Component (PC)	PC 1	PC 2
Eigenvalue	8.39	0.76
% variance	83.88	7.60
% cumulative variance	83.88	91.48

**Table 6:** The principal components and the factor coordinates of the variables.

Variables	PC 1	PC 2
Dry Matter	0.84	0.02
Proteins	−0.81	0.6
Crude Fat	−0.93	0.25
Ash	−0.89	−0.36
Total Carbohydrates	0.98	−0.15
Vitamin C	−0.96	0.24
Total Polyphenols	−0.94	−0.26
DPPH	−0.96	−0.23
Flavonoids	−0.96	−0.09
Phenolic acids	−0.88	−0.19
* Month	0.38	−0.64

* Supplementary variables.

## Data Availability

Not acceptable.

## Sample Availability

Samples of the plant material are not available from the authors.

## References

[1] Cardelle-Cobas A., Soria A.C., Corzo N., Villamiel M., Pacurar M., Krejci G. (2010). A Comprehensive Survey of Garlic Functionality. Garlic Consumption and Health.

[2] Arzanlou M., Bohlooli S. (2010). Introducing of green garlic plant as a new source of allicin. Food Chem..

[3] Dyduch J., Najda A. (2010). Yielding and quality of garlic leaves. Part I. Yield and its structure. EJPAU.

[4] González R.E., Sance M.M., Soto V.C., Galmarini C.R. (2012). Garlic scape, an alternative food with human health benefits. VI International Symposium on Edible Alliaceae.

[5] Oosthuizen C.B., Reid A.M., Lall N. (2018). Chapter 9––Garlic (Allium sativum) and its associated molecules, as medicine. Medicinal Plants for Holistic Health and Well-Being.

[6] Tavares L., Santos L., Pelayo C., Noreña Z. (2021). Bioactive compounds of garlic: A comprehensive review of encapsulation technologies, characterization of the encapsulated garlic compounds and their industrial applicability. Trends Food Sci. Technol..

[7] Przygoda B., Kunachowicz H., Nadolna I., Iwanow K. (2020). Nutritional Value of Selected Foods and Typical Dishes.

[8] Ciuba M., Dziadek K., Kukiełka E., Oczkowicz J., Piątkowska E., Leszczyńska T., Kopeć A. (2016). Comparing basic chemical composition and content of bioactive components in selected cultivars of garlic. Zywn-Nauk. Technol. Jakość.

[9] Ayaz E., Alpsoy H.C. (2007). Garlic (*Allium sativum*) and traditional medicine. Turk. Parazitolojii Dergisi..

[10] Martins N., Petropoulos S., Ferreira I.C. (2016). Chemical composition and bioactive compounds of garlic (*Allium sativum* L.) as affected by pre- and post-harvest conditions. Food Chem..

[11] Jędrszczyk E., Kopeć A., Bucki P., Ambroszczyk A.M., Skowera B. (2019). The enhancing effect of plants growth biostimulants in garlic cultivation on the chemical composition and level of bioactive compounds in the garlic leaves, stems and bulbs. Not. Bot. Horti Agrobot..

[12] Piątkowska E., Kopeć A., Leszczyńska T. (2015). Basic Chemical Composition, Content Of Micro and Macroelements And Antioxidant Activity Of Different Varieties Of Garlic’s Leaves Polish Origin. Zywn-Nauk. Technol. Jakość.

[13] Kuttan G. (2000). Immunomodulatory effect of some naturally occurring sulphur-containing compounds. J. Ethnopharmacol..

[14] Ruhee R.T., Roberts L.A., Ma S., Suzuki K. (2020). Organosulfur Compounds: A Review of Their Anti-inflammatory Effects in Human Health. Front. Nutr..

[15] Bastaki S.M.A., Ojha S., Kalasz H., Adeghate E. (2021). Chemical constituents and medicinal properties of Allium species. Mol. Cell Biochem..

[16] Sobenin I.A., Myasoedov V.A., Iltchuk M.I., Zhang D.W., Orekhovbe A.N. (2019). Therapeutic effects of garlic in cardiovascular atherosclerotic disease. Chin. J. Nat. Med..

[17] Gao X., Xue Z., Ma Q., Guo Q., Xing L., Kumar Santhanam R., Zhang M., Chen H. (2019). Antioxidant and antihypertensive effects of garlic protein and its hydrolysates and the related mechanism. J. Food Biochem..

[18] Ansary J., Forbes-Hernández T.Y., Gil E., Cianciosi D., Zhang J., Elexpuru-Zabaleta M., Simal-Gandara J., Giampieri F., Battino M. (2020). Potential Health Benefit of Garlic Based on Human Intervention Studies: A Brief Overview. Antioxidants.

[19] Donma M.M., Donma O. (2020). The effects of allium sativum on immunity within the scope of COVID-19 infection. Med. Hypotheses.

[20] Torres K.A.M., Lima S.M.R.R., Torres L.M.B., Gamberini M.T., Silva Junior P.I.D. (2021). Garlic: An Alternative Treatment for Group B Streptococcus. Microbiol. Spectr..

[21] Padiya R., Khatua T.N., Bagul P.K., Kuncha M., Banerjee S.K. (2011). Garlic improves insulin sensitivity and associated metabolic syndromes in fructose fed rats. Nutr. Metab..

[22] Kaur G., Padiya R., Adela R., Putcha U.K., Reddy G.S., Reddy B.R., Kumar K.P., Chakravarty S., Banerjee S.K. (2016). Garlic and Resveratrol Attenuate Diabetic Complications, Loss of β-Cells, Pancreatic and Hepatic Oxidative Stress in Streptozotocin-Induced Diabetic Rats. Front. Pharmacol..

[23] Ashfaq F., Ali Q., Haider M.A., Hafeez M.M., Malik A. (2021). Therapeutic activities of garlic constituent phytochemicals. Biol. Clin. Sci. Res. J..

[24] Cahayani A.W., Tanuwijaya C., Xiao Chi L., Mulyastuti Y. (2019). Antibacterial activity of garlic (*Allium sativum*) extract and molecular docking studies of allicin. AIP Conf. Proc..

[25] Hamal S., Cherukuri L., Birudaraju D., Matsumoto S., Kinninger A., Chaganti B.T., Flores F., Shaikh K., Roy S.K., Budoff M.J. (2020). Short-term impact of aged garlic extract on endothelial function in diabetes: A randomized, double-blind, placebo-controlled trial. Exp. Ther. Med..

[26] Ghyasi R., Mohaddes G., Naderi R. (2019). Combination effect of voluntary exercise and garlic (Allium sativum) on oxidative stress, cholesterol level and histopathology of heart tissue in type 1 diabetic rats. J. Cardiovasc. Thorac. Res..

[27] Oboh G., Ademiluyi A.O., Agunloye O.M., Ademosun A.O., Ogunsakin B.G. (2019). Inhibitory Effect of Garlic, Purple Onion, and White Onion on Key Enzymes Linked with Type 2 Diabetes and Hypertension. J. Diet Suppl..

[28] Zhang Y., Liua X., Ruan J., Zhuang X., Zhang X., Li Z. (2020). Phytochemicals of garlic: Promising candidates for cancer therapy. Biomed. Pharmacother..

[29] Silva L.A., Oliveira A.S., Melo F.L., Ardisson-Araújo D.M.P., Resende F.V., Resende R.O., Ribeiro B.M. (2019). A new virus found in garlic virus complex is a member of possible novel genus of the family *Betaflexiviridae* (order *Tymovirales*). PeerJ.

[30] Rekowska E., Skupień K. (2009). The influence of selected agronomic practices on the yield and chemical composition of winter garlic. Veg. Crops Res. Bull..

[31] Kosiorek A., Oszmiański J., Golański J. (2013). Rationale for the use of plant polyphenols as antiplatelet nutraceuticals. Post Fitoter..

[32] Haciseferoĝullari H., Őzcan M., Demir F., Calișir S. (2005). Some nutritional and technological properties of garlic (*Allium sativum* L.). J. Food Eng..

[33] Sajid M., Butt M.S., Shehzad A., Tanweer S. (2014). Chemical and mineral analysis of garlic: A golden herb. Pak. J. Food Sci..

[34] Marciniec K., Włodarczyk-Marciniec B. (2008). Anticancer properties of garlic. Post. Fitoter.

[35] Boonpeng S., Siripongvutikorn S., Sae-wong C., Sutthirak P. (2014). The antioxidant and anti-cadmium toxicity properties of garlic extracts. Food Sci. Nutr..

[36] Suleria H.A., Butt M.S., Khalid N., Sultan S., Raza A., Aleem M., Abbas M. (2015). Garlic (*Allium sativum*): Diet based therapy of 21st century: A review. Asian Pac. J. Trop. Dis..

[37] Yusuf A., Fagbuaro S.S., Fajemilehin S.O.K. (2018). Chemical composition, phytochemical and mineral profile of garlic (*Allium sativum*). J. Biosci. Biotechnol. Discov..

[38] Różańska D., Regulska-Ilow B., Ilow R. (2014). Influence of selected culinary processes on the antioxidant capacity and polyphenol content in food. Probl. Hig. Epidemiol..

[39] Mnayer D., Fabiano-Tixier A., Petitcolas E., Hamieh T., Nehme N., Ferrant C., Fernandez X., Chemat F. (2014). Chemical composition, antibacterial and antioxidant activities of six essentials oils from the Alliaceae family. Molecules.

[40] Leelarungrayub N., Rattanapanone V., Chanarat N., Gebicki J.M. (2006). Quantitative evaluation of the antioxidant properties of garlic and shallot preparations. Nutrition.

[41] Queiroz Y.S., Ishimoto E.Y., Bastos D.H.M., Sampaio G.R., Torres E.A.F.S. (2009). Garlic (*Allium sativum* L.) and ready-to-eat garlic products: In vitro antioxidant activity. Food Chem..

[42] Kim J., Pälijärvi M., Karonen M., Salminen J.P. (2020). Distribution of enzymatic and alkaline oxidative activities of phenolic compounds in plants. Phytochemistry.

[43] Kim J.S., Kang O.J., Gweon O.C. (2013). Comparison of phenolic acids and flavonoids in black garlic at different thermal processing steps. J. Funct. Foods.

[44] Miean K.H., Mohamed S. (2001). Flavonoid (myricetin, quercetin, kaempferol, luteolin, and apigenin) content of edible tropical plants. J. Agric. Food Chem..

[45] Gorinstein S., Leontowicz H., Leontowicz M., Namiesnik J., Najman K., Drzewiecki J., Cvikrova M., Martincova O., Katrich E., Trakhtenberg S. (2008). Comparison of the main bioactive compounds and antioxidant activities in garlic and white and red bulbs after treatment protocols. J. Agric. Food Chem..

[46] Strugała P., Gabrielska J. (2014). Biological activity and stability in vitro of polyphenolic extracts as potential dietary supplements. Postepy. Hig. Med. Dosw..

[47] Callemien D., Collin S. (2009). Structure, organoleptic properties, quantification methods, and stability of phenolic compounds in beer—A Review. Food Rev. Int..

[48] Palma M., Piñeiro Z., Barroso C.G. (2001). Stability of phenolic compounds during extraction with superheated solvents. J. Chromatog. A.

[49] Ghareaghajlou N., Hallaj-Nezhadi S., Ghasempour Z. (2021). Red cabbage anthocyanins: Stability, extraction, biological activities and applications in food systems. Food Chem..

[50] Patras A., Brunton N.P., O’Donnell C., Tiwari B.K. (2010). Effect of thermal processing on anthocyanin stability in foods; mechanisms and kinetics of degradation. Trends Food Sci. Technol..

[51] Nenadis N., Wang L.F., Tsimidou M., Zhang H.Y. (2004). Estimation of scavenging activity of phenolic compounds using the ABTS(*+) assay. J. Agric. Food Chem..

[52] Al-Qudah M.A., Migdadi R.S., Mayyas A.S., Al-Zereini W.A., Al-Dalahmeh Y., Abu Orabi F.M., Bataineh T.T., Abu-Orabi S.T. (2022). Chemical Composition, Cytotoxicity and Antioxidant Activity of the Essential Oil from Flower Buds and Leaves of the Pulicariaincisa (Lam.) DC and *Pulicaria crispa* (Forskel) Oliver. J. Essent. Oil Bear. Plants.

[53] Arnao M.B. (2000). Some methodological problems in the determination of antioxidant activity using chromogen radicals: A practical case. Trends Food Sci. Technol..

[54] AOAC International (2006). AOAC International: Official Methods of Analysis.

[55] Metzger L.E., Nielsen S.S., Nielsen S.S. (2017). Nutrition labeling in food analysis. Food Analysis.

[56] Rutkowska U. (1981). Selected Methods in Measuremnts of Chemical Composition and Nutritional Value of Food Products.

[57] Swain P., Hillis W.E. (1959). The phenolic constituents of *Prunus domestica* (L.). The quantity of analisys of phenolic constituents. J. Sci. Food Agric..

[58] Re R., Pellegrini N., Proteggente A., Pannala A., Yang M., Rice-Evans C. (1999). Antioxidant activity applying an improved ABTS radical cation decolorization assay. Free Radic. Biol. Med..

[59] Pekkarinen S.S., Stockmann H., Schwarz K., Heinonen I.M., Hopia A.I. (1999). Antoxidant activity and partitioning of phenolic acids in bulk and emulsifed methyl linoleate. J. Agric. Food Chem..

[60] Benzie I.F.F., Strain J.J. (1996). The Ferric Reducing Ability of Plasma (FRAP) as a Measure of “Antioxidant Power”: The FRAP Assay. Anal. Biochem..

[61] Dziadek K., Kopeć A., Tabaszewska M. (2019). Potential of sweet cherry (*Prunus avium* L.) by-products: Bioactive compounds and antioxidant activity of leaves and petioles. Eur. Food Res. Technol..

[62] Wallock-Richards D., Doherty C.J., Doherty L., Clarke D.J., Place M., Govan J.R.W., Campopano D.J. (2014). Garlic revisited: Antimicrobial activity of allicin-containing garlic extracts against *Burkholderia cepacia* complex. PLoS ONE.

[63] Sadowska U., Jewiarz K., Kopak M., Dziadek K., Francik R., Kopeć A. (2023). Proximate analysis and antioxidant properties and of young plants of *Sinapis alba* L. depend on the time of harvest and variety. Appl. Sci..

[64] Jolliffe I.T. (2002). Principal Component Analysis.

[65] Catell R.B. (1996). The Scree Test for the Number of Factors. Multivar. Behav. Res..

